# Isolation and identification of culturable bacteria from wild *Anopheles culicifacies*, a first step in a paratransgenesis approach

**DOI:** 10.1186/1756-3305-7-419

**Published:** 2014-09-04

**Authors:** Ali Reza Chavshin, Mohammad Ali Oshaghi, Hasan Vatandoost, Mohammad Reza Pourmand, Ahmad Raeisi, Olle Terenius

**Affiliations:** Social Determinants of Health, Research Center, Urmia University of Medical Sciences, Urmia, Iran; Department of Medical Entomology and Vector Control, School of Public Health, Urmia University of Medical Sciences, Urmia, Iran; Department of Medical Entomology and Vector Control, School of Public Health, Tehran University of Medical Sciences, Tehran, Iran; Department of Medical Biotechnology, School of Advanced Medical Technology, Tehran University of Medical Sciences, Tehran, Iran; Department of Ecology, Swedish University of Agricultural Sciences (SLU), Uppsala, Sweden

**Keywords:** Midgut microbiota, 16S rRNA, *Anopheles culicifacies*, Malaria, Paratransgenesis

## Abstract

**Background:**

Due to the effect of midgut bacteria on proliferation of parasites and their potential as paratransgenesis tools, their identification in malaria vector mosquitoes is important. *Anopheles culicifacies* s.l. is one of the main malaria vectors in Asia; however, its midgut microbiota remains un-studied. This work was primarily designed to isolate potential candidates for use in a paratransgenesis approach, but also to give a picture of the midgut microbiota of wild-caught *An. culicifacies* larvae and adults from the southeast corner of Iran, which has the highest malaria endemicity in the country.

**Methods:**

A total of 68 larvae and 34 adult females (newly eclosed and older) from three different biotopes in Iran were analyzed for their midgut microflora. The mosquitoes had their midgut bacterial contents plated on three different culture media (brain heart agar, nutrient agar and blood agar) yielding 57 bacterial isolates. The 16S rRNA genes of the isolates were sequence analyzed for species designation, which then was confirmed by biochemical analysis.

**Results:**

A total of twelve bacterial genera were identified: *Acinetobacter*, *Aeromonas*, *Bacillus*, *Chryseobacterium*, *Delftia*, *Exiguobacterium*, *Kurthia*, *Microbacterium*, *Pseudomonas*, *Staphylococcus*, *Thorsellia* and *Variovorax*. In older females, only Gram-negative bacteria were found, whereas larvae and newly-eclosed adults also harbored Gram-positive bacteria. The diversity of isolates also varied between sampling sites and mosquito stages, with the largest number of genera found in the Anguri district and in larvae, respectively. *Pseudomonas* was the most common genus retrieved from all sampling sites, and in both larvae and adults, suggesting a potential transstadial passage of these bacteria. Interestingly, identical 16S sequences of *Pseudomonas* were found in mosquitoes originating from different habitats at least 45 km apart, which could suggest that these bacteria have been adapted to the mosquitoes.

**Conclusions:**

The study of vector mosquito microbiota has recently gathered increased interest because of the potential influence on vector competence. By adding data from a hitherto uncharacterized malaria mosquito, a better picture of gut flora in vector mosquitoes was obtained. Furthermore, some species of the predominant genus *Pseudomonas* will be evaluated for the selection of a paratransgenesis candidate.

## Background

Vector-borne diseases cause health problems in several parts of the world and among them malaria is the most important with almost 700,000 deaths annually [1]. Malaria control programs focused on mosquito vectors have reduced mortality and morbidity, but emerging insecticide-resistant vectors and environmental issues related to application of pesticides have necessitated development of new control strategies with less environmental impacts/damage and higher efficacy [2, 3].

One of the control approaches under development is paratransgenesis, which has been defined as using symbiotic organisms (such as bacteria) to deliver anti-parasitic effector molecules to wild vector populations [4]. Initial steps of developing paratransgenesis against malaria have been taken in laboratory experiments on *An. gambiae* mosquitoes. In an early study, a single-chain antibody targeting *Plasmodium berghei* ookinete Pbs21 was linked to the lytic peptide Shiva-1 and expressed in *Escherichia coli*, which resulted in 95.6% transmission blockage [5]. More recently, it was shown that engineered *Pantoea agglomerans* bacteria isolated from mosquitoes inhibited development of the human malaria parasite *Plasmodium falciparum* and the rodent malaria parasite *Plasmodium berghei* by up to 98% [6].

In mosquitoes, obligate symbionts are yet to be found; therefore, a first step in paratransgenesis is to identify the normal midgut microbiota of mosquitoes and to isolate candidates for further modification [7, 8]. So far, a limited number of studies have been carried out on the microbiota of *Anopheles* mosquitoes [9]. The studied species include: laboratory-reared *Anopheles stephensi*, *An. gambiae*, and *An. albimanus*[10], field-collected *An. albimanus* from southern Mexico [11], field-collected *An. gambiae* s.s., *An. arabiensis* and *An. funestus* from western Kenya [12], field-collected *An. darlingi* from Brazil [13], laboratory-reared and wild-caught *An. stephensi* from India [14], semi-field collected *An. gambiae* from Kenya [15], field-collected *An. maculipennis* and *An. stephensi* from Iran [16], and field-collected *An. stephensi* from Iran [17].

*An. culicifacies* Giles is one of the main malaria vectors in the tropical parts of South and Southwest Asia [18–23], where it transmits both *Plasmodium falciparum* and *P. vivax*[24]. In this study, culturable midgut bacteria isolated from wild-caught *An. culicifacies* in southeastern Iran are identified using 16S rRNA sequence analysis with the aim of selecting potential candidates for paratransgenesis.

## Methods

### Ethics statement

Prior to the approval of all projects by the Tehran University of Medical Sciences (TUMS), they are reviewed and endorsed by the ethical committee of the TUMS. Mosquito collection was carried out from private dwellings. At least one week prior to any mosquito collection, the owners were informed by the Local Health System officers. The research and its objectives were explained by Ali Reza Chavshin (ARC), to residents. Owners of the land (for larval collection) and dwellings (for adult mosquito collection) gave permission to conduct the study on these sites. After their permission, the samples were collected at an agreed date and time. The whole process was coordinated, managed and documented by the “Local manager of malaria control program” and “Local Health System officer” in the study areas and signed by ARC. Also it is declared that the collected species is not endangered or protected in the area of investigation.

### Field collection of *An. culicifacies*and isolation of midgut bacteria

**Figure 1:**
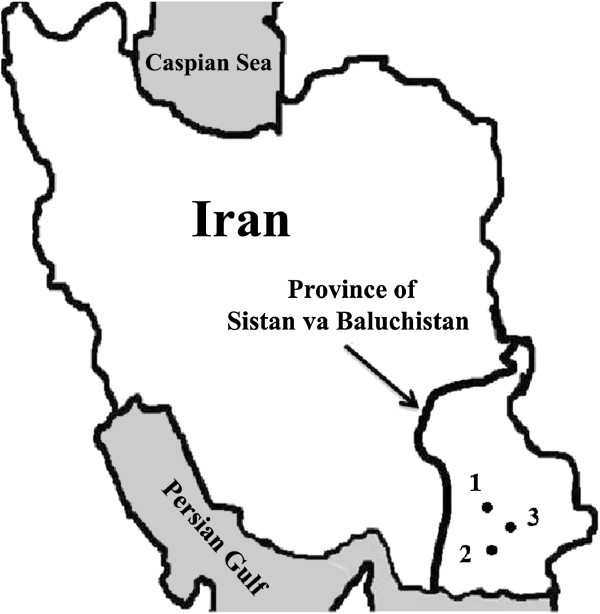
**Map of Iran indicating the locations of the Sistan-va-Baluchistan Province**, **and the districts/villages where the specimens were collected.** 1) Iranshahr, 2) Anguri, and 3) Saraydan.

Wild caught mosquito samples were collected during July-September 2010, from 1) the Iranshahr district, an urban region; 2) the Anguri district, a mountainous or hilly rural region with temporal rivers; and 3) the Saraydan district, a rural plain region (Table 1). The distances between the three sites are 45–90 km (Iranshahr to Saraydan 45 km, Saraydan to Anguri 50 km, and Iranshahr to Anguri 90 km). All three areas are parts of the Sistan and Balouchistan Province, which borders to Pakistan and comprises the most important urban and rural malaria foci in southeastern Iran (Figure 1).

**Table 1:** **The geographical and ecological properties of the sampling sites**

Region	Geographical location		Zone	Vegetation	Collected samples	
	Latitude (°N)	Longitude (°E)			Larvae	Adult females
Iranshahr	27° 12' 27.94"	60° 39' 54.42"	Urban	Palm	25	11
Anguri	26° 40' 51.24"	61° 12' 47.99"	Rural	Palm and fruit trees	18	9
Saraydan	27° 7' 46.82"	60° 52' 32.10"	Rural	Rice field and fruit trees	25	14

A standard dipping technique was used for larval sample collection (350 ml dipper). Adult mosquitoes were collected by aspiration from the walls and the roofs of dwellings and pit shelters where adults normally rest after feeding [25]. The live specimens were transferred to the laboratory of Iranshahr, National Institute of Health Research (NIHR). All collected samples were identified to species level using standard morphological keys [26]. In this part of Iran, *An. culicifacies* subspecies A is the most abundant [27]. Among the identified species, the *An. culicifacies* s.l. were separated and analyzed for midgut microbiota. Specimens analyzed for midgut microbiota were from three sources: 1) wild-caught females, 2) wild-caught fourth instar larvae, and 3) newly emerged female adults (immediately dissected after emergence from pupa reared from a subset of the larvae in 2).

Preparation, sterilization and dissection of specimens were done according to a previously described method [28]. Obtained midguts were mashed and suspended in 500 μL of Brain Heart Infusion (BHI). A 100 μL aliquot of the contents was serially diluted up to 10^-6^ and plated onto different media: 1) Brain Heart agar (BHA), 2) Nutrient agar (NA), and 3) Blood agar (BA) (Merck, Germany) and incubated at 28 ± 2°C for 24-48 hours. The sterility of all reagents was checked and controls for the efficiency of sterilization were treated like the other samples. Continuous sub-culture of every grown bacterial colony was performed in order to isolate single purified colonies of the bacteria. The single colonies of the bacteria were later used for DNA extraction and PCR, and biochemical investigations.

### DNA extraction, 16S rRNA gene amplification and sequencing

Each purified bacterial colony was subjected to genomic DNA extraction using the QIAGEN DNeasy Kit (Qiagen, Germany) according to the manufacturer’s instructions. The 16S rRNA universal primers 16suF 5′-GAGTTTGATCCTGGCTCAG-3′ and 16suR 5′-GTTACCTTGTTACGACTT-3′ [29] were used to amplify about 1500 bp long sequences. The PCR program had an initial denaturation at 94°C for 10 min, followed by 35 cycles of denaturation at 95°C for 30 s, annealing at 56.5°C for 40 s, and extension at 72°C for 30 s, followed by a final extension at 72°C for 8 min. All amplicons were sent for sequencing to SeqLab (Germany). The Mallard program (http://www.bioinformatics-toolkit.org) was used for all acquired sequences to check the presence of probable chimeric sequences and the specimens with suspicious sequences were removed from the data set. The resultant sequences were compared to the data-bases of the Ribosomal Database Project (RDP II; Michigan State University, http://rdp.cme.msu.edu) and the GenBank (http//:http://www.ncbi.nlm.nih.gov/BLAST) for confident sequence analysis and their seq-match and sequences similarity check tools were used.

**Figure 2:**
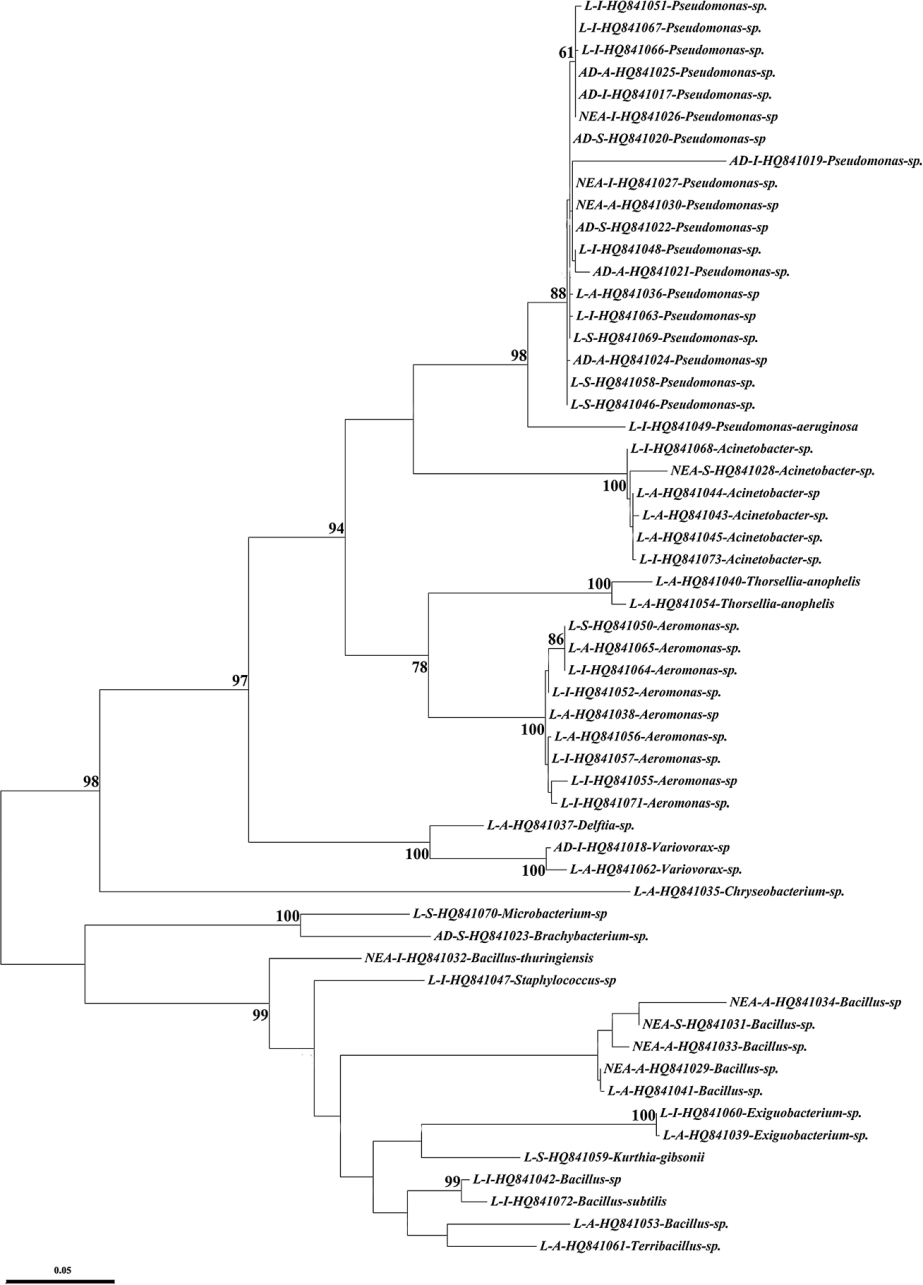
**Phylogenetic analysis of 16S rRNA sequences (~1500 bp) of all bacteria isolated from** ***An. culicifacies*** **using Maximum Likelihood (ML) based on the Tamura 3-parameter model**[33]. The first part of the sequence ID indicates from which life stage the bacteria were isolated, adult (A), newly emerged adult (NEA), or larva (L). The second part of the sequence ID denotes the sampling locality, Anguri (A), Iranshahr (I), or Saraydan (S). The third part of the sequence ID is the GenBank accession number. Only the significant bootstrap values (>50%) from 1000 replicates are shown on the nodes.

Based on sequence comparison with the GenBank and RDPII entries, identification of isolates and their classification at genus and species level was done; 99 percent or higher sequence identities with the GenBank entries were assumed for species delineation [30]. All isolates were also identified using classical phenotyping and biochemical methods such as Gram-staining, oxi/ferm tests and using selective cultivation media [31]. The results of biochemical and phenotyping methods were compared to the sequencing results and only those bacteria that confirmed the sequencing results are presented. All confirmed sequences were submitted to GenBank. All GenBank reference numbers are displayed in Figure 2.

The MEGA5.05 [32] software was used for phylogenetic analysis and tree construction. Phylogenetic trees were built using Maximum Likelihood (ML) based on the Tamura 3-parameter model [33] (1000 bootstrap replicates) analyses.

## Results

In this study, midguts of a total of 102 specimens of *An. culicifacies* (68 larvae and 34 adults) from three different sampling sites were analyzed (Table 1). The specimens were dissected and screened for cell-free cultivable bacteria on different media resulting in a total of 57 bacterial isolates (Table 2). Forty-one isolates were members of seven genera of Gram-negative bacteria and the remaining 16 isolates belonged to Gram-positive bacteria. Gram-negative bacteria were isolated from larvae and both adult stages, but Gram-positive bacteria were only isolated from larvae and newly emerged adults. The domination of Gram-negative bacteria was statistically significant in larvae and in mosquitoes from the Iranshahr area (Table 3). Sequence data of the bacteria that were found in the midgut of larval and adult stages were used for phylogenetic analysis.

**Table 2:** **The number of purified colonies from midguts of** ***An. culicifacies*** **in relation to the culture media and life stage of mosquitoes**

Culture medium	Life stage of dissected ***An. culicifacies***	Number of purified colonies
BH agar	Larva	12
	Newly emerged adult^a^	6
	Adult	5
Nutrient agar	Larva	12
	Newly emerged adult^a^	4
	Adult	4
Blood Agar	Larva	14
	Adult	0

^a^Dissected immediately after emergence.

**Table 3:** **Number of Gram**-**negative and Gram**-**positive isolates in different mosquito life stages and areas**

Stage	Gram-negative	Gram-positive	p-value (Chi-test)
Larva	29	7	0.00025
Newly emerged adult^a^	4	6	N/A
Adult	8		N/A
Area			
Anguri	16	7	0.061
Iranshahr	18	5	0.0067
Saraydan	7	4	N/A

^a^Dissected immediately after emergence.

A total of 19 bacterial species were isolated and identified from the different stages of the collected *An. culicifacies* specimens. In Figure 2, all non-identical 16S sequences are represented with information on stage and sampling locality. According to the number of isolated and identified species, it was shown that the larval stage of the wild-caught samples had a higher diversity with 17 species divided in 12 genera of bacteria: *Acinetobacter*, *Aeromonas*, *Bacillus*, *Chryseobacterium*, *Delftia*, *Exiguobacterium*, *Kurthia*, *Microbacterium*, *Pseudomonas*, *Staphylococcus*, *Thorsellia* and *Variovorax*. In contrast, in wild-caught adults only three species from three genera were identified: *Brachybacterium*, *Pseudomonas* and *Variovorax*. In the newly emerged adults from wild caught larvae four species from three genera of bacteria were identified: *Acinetobacter*, *Bacillus*, and *Pseudomonas* (Table 4).

**Table 4:** **The isolated genera of bacteria from the midgut of** ***An. culicifacies*** **in relation to mosquito life stage and sampling locations**

Region	Larvae	Newly emerged adults	Adult females
Iranshahr	*Acinetobacter*, *Aeromonas*, *Bacillus*, *Exiguobacterium*, *Pseudomonas*, *Staphylococcus*	*Bacillus*, *Pseudomonas*	*Pseudomonas*, *Variovorax*
Anguri	*Acinetobacter*, *Aeromonas*, *Bacillus*, *Chryseobacterium*, *Delftia*, *Exiguobacterium*, *Pseudomonas*, *Thorsellia*, *Variovorax*	*Bacillus*, *Pseudomonas*	*Pseudomonas*
Saraydan	*Aeromonas*, *Kurthia*, *Microbacterium*, *Pseudomonas*	*Acinetobacter*, *Bacillus*	*Brachybacterium*, *Pseudomonas*

**Figure 3:**
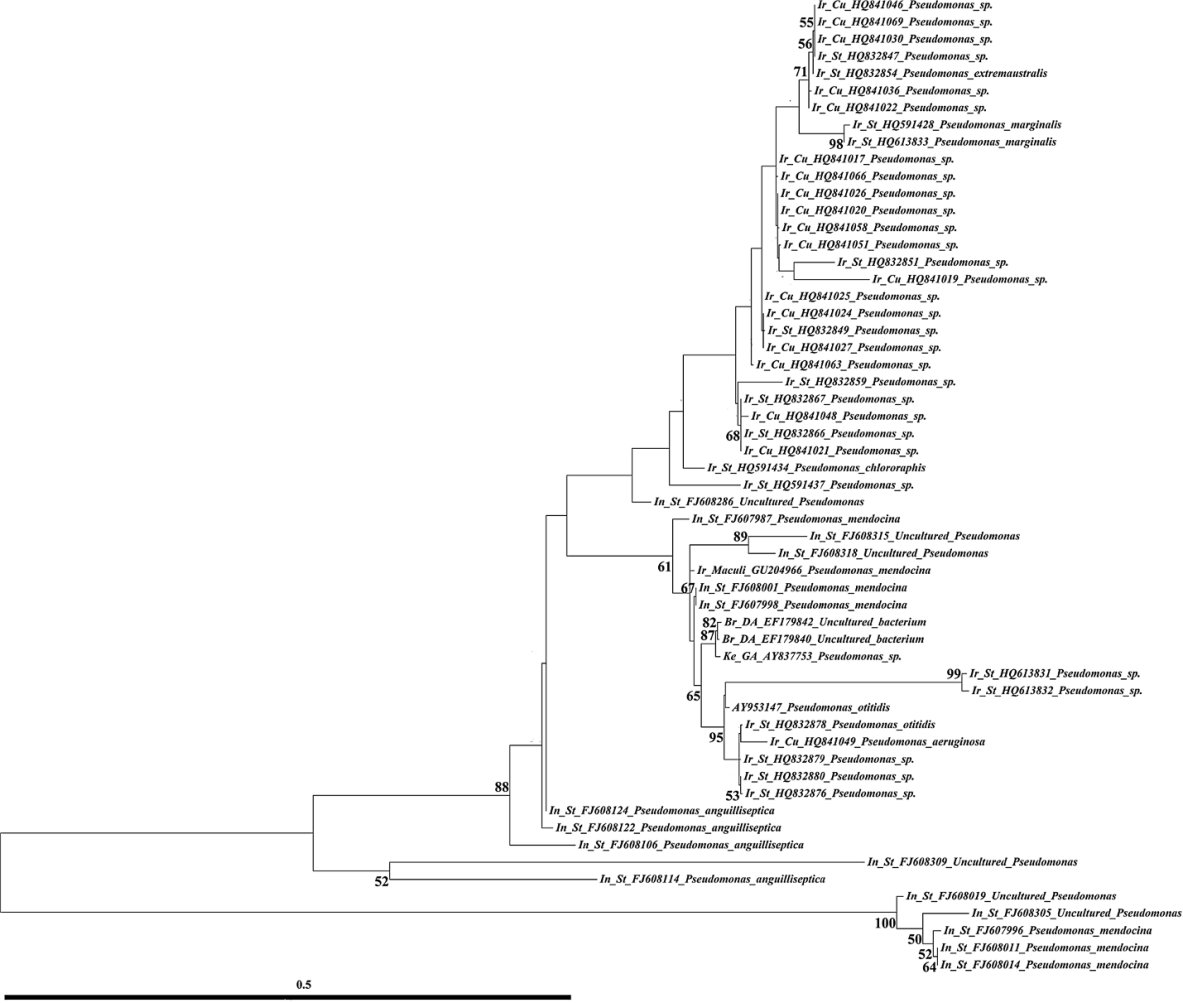
**Phylogenetic analysis of 16S rRNA sequences (~1500 bp) of** ***Pseudomonas*** **strains using Maximum Likelihood (ML) based on the Tamura 3-parameter model**[33]**from different** ***Anopheles*** **mosquitoes:** ***An. culicifacies*** **from Iran (Ir_Cu; this study),** ***An. darlingi*** **from Brazil (Br_DA; [**[13]**]),** ***An. gambiae*** **from Kenya (Ke_GA; [**[12]**]),** ***An. stephensi*** **from India (In_St;** **[**[14]**]),** ***An. stephensi*** **from Iran (Ir_St; [**[17]**]) and** ***An. maculipennis*** **from Iran (Ir_Maculi; [**[16]**]).** Only the significant bootstrap values ( >50%) are shown on the nodes. Scale of genetic distance is shown underneath.

The genus *Pseudomonas* was the most frequently isolated bacteria in this study (20/57, 57%) and members of *Pseudomonas* have been also identified in other malaria vectors [10, 13, 14, 16, 17]. To show the relationship between sequence data and origin of samples, all the sequences belonging to the genus *Pseudomonas* from *An. darlingi*, *An. gambiae*, *An. maculipennis* and *An. stephensi* available in GenBank were retrieved and analyzed together with the ones found for *An. culicifacies* in this study (Figure 3).

## Discussion

Identification of gut bacteria in vector mosquitoes has received notable attention since recent studies suggest that the composition of the vector gut microbiota affects the outcome of mosquito infection with *Plasmodium* parasites [34, 35]. This corroborates earlier results, for example that some bacteria have a natural anti-*Plasmodium* effect [10, 11, 36]. Although a number of studies have been carried out to identify the bacterial microbiota of *Anopheles* mosquitoes, several important vectors remain un-studied. Among those was *An. culicifacies*, which has been investigated in the current study, and from which aerobic midgut bacteria have been isolated and identified. Strikingly, as seen by the lack of branch length differences between the isolates in Figure 2, many are very similar despite coming from different stages and different areas. This suggests that the bacteria acquired by *An. culicifacies* is not a random selection, but instead points to possible co-adaptation between bacteria and mosquitoes and/or their breeding waters.

Albeit the number of isolates in this study is moderate, the data suggest that Gram-negative bacteria dominate the flora of *An. culicifacies* (Table 3). This agrees with earlier results from culture-based studies on *An. stephensi* and *An. maculipennis* in Iran [16] and *An. gambiae* and *An. funestus* in Kenya and Mali [37], and also data from sequence-based studies on *An. darlingi* in Brazil [13] and *An. gambiae* in Kenya [15] all showing predominance for Gram-negative bacteria. In Wang *et al*. [15], the proportion of Gram-negative bacteria increases from larvae to adults, similar to what we see for *An. culicifacies*.

Comparing the results of this study with other similar studies showed that some bacteria are common among several important vectors. For instance the genus *Thorsellia*, which was isolated from *An. arabiensis*[12] and *An. stephensi*[14] and also found in *An. gambiae* s.l. [15, 38], has now been identified in *An. culicifacies* (this study). In two different Kenyan populations of *An. gambiae* mosquitoes, *Thorsellia* was the dominant genus [15, 38]. Briones *et al*. [38] consistently isolated *T. anophelis* from the water surface micro-layer (SML), i.e., where *Anopheles* larvae feed, as well as in 40% of the adults, while Wang *et al*. [15] found that almost 70% of the bacteria in young adults belonged to the *Thorsellia* genus. *Thorsellia* is adapted to a life in mosquitoes with high tolerance for the alkaline conditions found in the larvae and with increased growth rates in blood medium [38]. *Thorsellia* has only been found in vector mosquitoes and their breeding waters, and appears to be a unique genus of bacteria only distantly related to other members of gamma-proteobacteria [39].

Another common bacterial genus is *Pseudomonas*, which has been found among several mosquito vectors in Asia and the Americas [11, 13, 14, 17, 36]; however, a recent study found *Pseudomonas* only at a low level in Kenyan mosquitoes [40]. In this study, we identified *Pseudomonas* as the most frequently isolated bacteria from *An. culicifacies*. The same finding was made previously in *An. stephensi* midguts [17]. It should be noted that although *Pseudomonas* is the most frequently isolated species, other non-culturable species may be important constituents of the midgut microbiota in *An. culicifacies*. However, even in studies on mosquito gut flora mainly using PCR-based methods for identification, those bacteria possible to grow under laboratory conditions dominate the gut flora [13, 15, 40]. The high frequency of *Pseudomonas* isolates is promising for a paratransgenesis approach also because of its possibility to grow in cell-free and ordinary culture media and suitability for genetic transformation [41]. However, characteristics such as transstadial transmission, non-pathogenicity, immunological and physiological adaptation to mosquito midgut conditions, colonization in the mosquito midgut including effective competition with resident bacteria and persistency in the gut for a reasonable time, should be studied before selection of the isolates for paratransgenesis. A note of caution is that among the more than 100 *Pseudomonas* species some are pathogenic; for example, *Pseudomonas aeruginosa* is an opportunistic human pathogen in severely immunocompromised patients [42]. Therefore, care must be taken so that for a paratransgenesis approach only *Pseudomonas* species that are non-pathogenic should be selected.

In a paratransgenesis strategy against mosquito-borne diseases, it would be an advantage if the transformed bacteria expressing effector molecules could remain in the vector population. Some kind of transstadial transmission would therefore be an essential characteristic for an ideal paratransgenesis candidate. However, it was suggested that most bacteria are lost during metamorphosis in the pupal stage [43]. In this study, the sequence and phylogenetic analysis showed that some isolates of the genus *Pseudomonas* were common and present in field-collected larvae, adults, and newly emerged adults from field-collected larvae. Some of the *Pseudomonas* isolates found in different stages of *An. culicifacies* from the same area have 16S sequences with 100% sequence identity suggesting transstadial transmission. This finding is similar to a possible transstadial transmission of some *Pseudomonas* isolates in *An. stephensi*[17], but needs to be further investigated.

## Conclusions

This is the first study on microbiota in *An. culicifacies*, an important vector of malaria in Asia. Further studies are needed regarding the biological characteristics of the bacteria and interactions between the gut microbiota and the host. The fact that *Pseudomonas* bacteria with identical 16S sequences are present both in different locations and different stages could suggest that this species has adapted to a life in *An. culicifacies*. The potential symbiotic relationship between *An. culicifacies* and *Pseudomonas* makes it a good candidate for paratransgenesis.
